# Sequence-specific RNA cleavage by PNA conjugates of the metal-free artificial ribonuclease tris(2-aminobenzimidazole)

**DOI:** 10.3762/bjoc.11.55

**Published:** 2015-04-16

**Authors:** Friederike Danneberg, Alice Ghidini, Plamena Dogandzhiyski, Elisabeth Kalden, Roger Strömberg, Michael W Göbel

**Affiliations:** 1 Institute for Organic Chemistry and Chemical Biology, Goethe Universität Frankfurt, Max-von-Laue-Straße 7, D-60438 Frankfurt am Main, Germany; 2 Karolinska Institutet, Department of Biosciences and Nutrition, Novum, SE-141 83 Huddinge, Sweden

**Keywords:** antisense, fluorescence correlation spectroscopy, guanidine, miRNA 20a, peptide nucleic acids

## Abstract

Tris(2-aminobenzimidazole) conjugates with antisense oligonucleotides are effective site-specific RNA cleavers. Their mechanism of action is independent of metal ions. Here we investigate conjugates with peptide nucleic acids (PNA). RNA degradation occurs with similar rates and substrate specificities as in experiments with DNA conjugates we performed earlier. Although aggregation phenomena are observed in some cases, proper substrate recognition is not compromised. While our previous synthesis of 2-aminobenzimidazoles required an HgO induced cyclization step, a mercury free variant is described herein.

## Introduction

Sequence specific artificial ribonucleases are an attractive research target for several reasons. On the one hand, they can improve our mechanistic understanding of natural ribonucleases and of phosphodiester reactivity in general. On the other hand, a wide range of practical applications is conceivable for such RNA cleavers ranging from tools in molecular biology to chemotherapeutics targeting mRNAs or miRNAs. Although most site-selective artificial ribonucleases consist of a catalytic unit (both with and without metal ions) attached to a DNA oligonucleotide or 2’-*O*-methyloligoribonucleotide complementary to the targeted RNA [1–3], possible future in cell applications suggest the conjugation of RNA cleavers to oligonucleotide analogues such as peptide nucleic acids (PNA) [4]. The benefits of oligonucleotide analogues are higher affinity towards RNA [5] as well as improved resistance against biodegradation [6]. Furthermore, PNA can be applied to block miRNA functions in cell culture experiments [7].

**Scheme 1:**
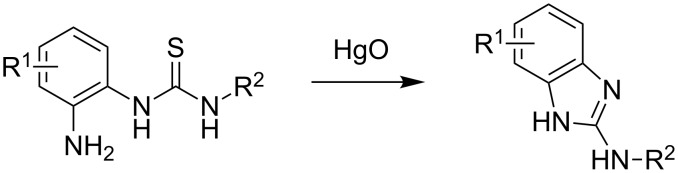
Formation of the 2-aminobenzimidazole moiety.

PNA conjugates of both metal-containing and metal-free RNA cleavers have been effectively used as artificial nucleases towards different substrates [8–11]. Most notable is a copper-based PNAzyme which cleaves RNA site-specifically with substrate half-lifes as low as 30 minutes [12]. A PNA-10mer attached to diethylenetriamine showed a higher cleavage activity [13] when compared to the corresponding 22mer DNA conjugate [14]. We therefore decided to investigate the activity of tris(2-aminobenzimidazole) [15] – a metal-free ribonuclease developed by us formerly – as a conjugate with PNA. Previous results for the corresponding DNA conjugates had shown sequence specific RNA cleavage with substrate half-lives in the range of 12 to 17 h [16]. Tris(2-aminobenzimidazole) **7** can be obtained in a six-step reaction sequence starting from tris(2-aminoethyl)amine (**1**) [15]. However, the use of toxic mercury(II) oxide for the formation of the benzimidazole moieties (Scheme 1) is a disadvantage of this synthesis especially with regard to future biological applications of the final product. Furthermore, conditions for these reactions are rather harsh and the yields may differ considerably. Here we report a new and mercury-free synthesis of tris(2-aminobenzimidazole) and its conjugation to PNA oligomers. In addition, the results of cleaving experiments with three different RNA substrates are presented.

## Results and Discussion

Alternative reagents to convert thioureas into guanidines include metal salts like CuCl_2_ or HgCl_2_ and carbodiimides such as 1-ethyl-3-(3-dimethylaminopropyl)carbodiimide (EDCI) or *N,N’*-diisopropylcarbodiimide (DIC) [17]. Another method developed by Lipton et al. uses Mukaiyama's reagent (2-chloro-1-methylpyridinium iodide) to prepare guanidines in high yields [18]. The reaction proceeds at ambient temperature. In contrast to ureas and thioureas formed as byproducts when using carbodiimides, the *N*-methylpyridine-2(1*H*)-thione resulting from Mukaiyama's reagent is soluble and can be easily removed by chromatography. As we were searching for a heavy metal-free approach towards tris(2-aminobenzimidazole), we adopted Lipton's method to the synthesis of aminobenzimidazoles.

**Scheme 2:**
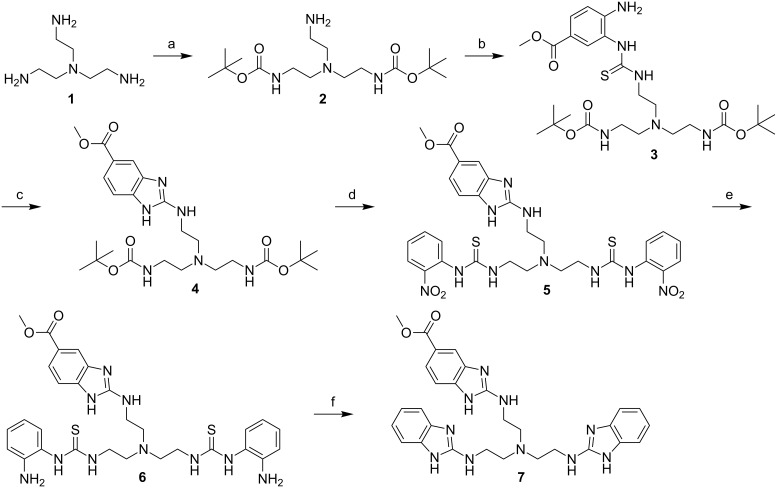
Synthesis of tris(2-aminobenzimidazole). Conditions: a: Boc-ON, THF, 0 °C to rt, 46 h, 45%; b: 1) 1,1‘-thiocarbonyldiimidazole, imidazole, MeCN, 0 °C to rt, 1 h; 2) methyl 3,4-diaminobenzoate, MeCN, 50 °C, 4 h, rt, overnight, 79%; c: Mukaiyama’s reagent, NEt_3_, DMF, 20 h, rt, 88%; d: 1) SOCl_2_, MeOH, 0 °C to rt, 3 h; 2) 2-nitrophenylisothiocyanate, NEt_3_, EtOH, 0 °C to rt, overnight, 90%; e: 45 bar H_2_, Pd/C (10%), MeOH sat. with NH_3_, 60 °C, 5 h, rt, overnight, 37%; f: Mukaiyama’s reagent, NEt_3_, DMF, 22 h, rt, 56%. Boc-ON: 2-(*tert*-butoxycarbonyloxyimino)-2-phenylacetonitrile.

**Scheme 3:**
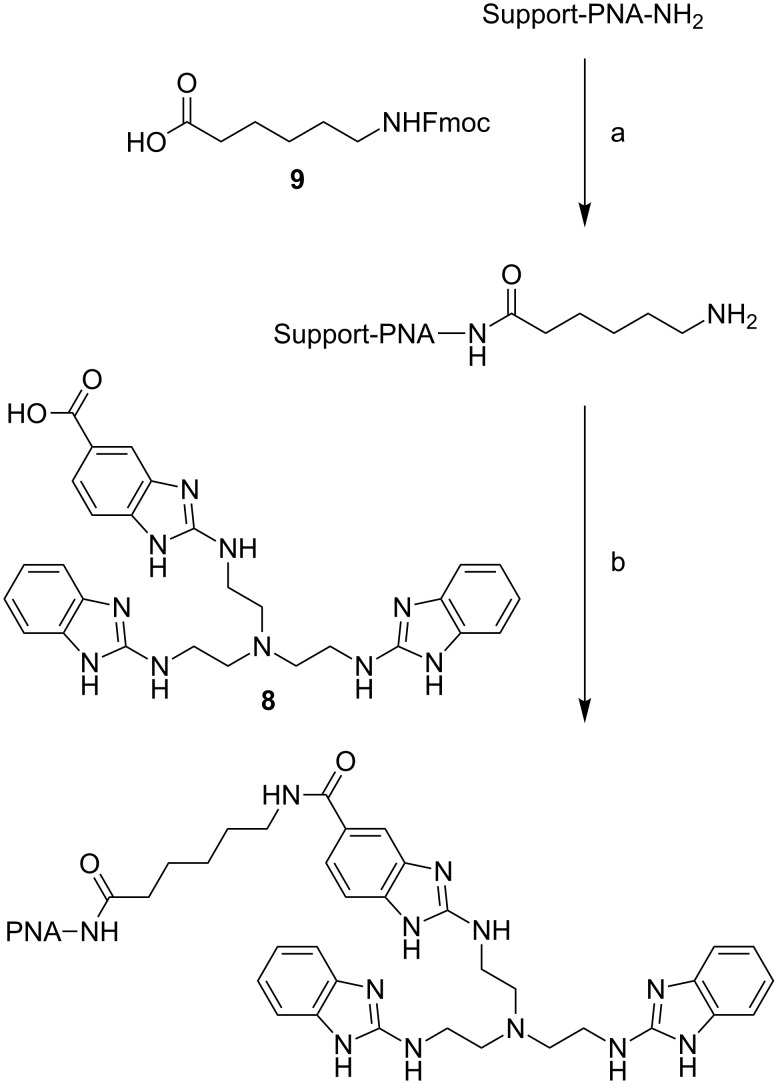
Synthesis of PNA conjugates. Conditions: a: 1) **9**, HOBt, DIC, DMF, rt, 24 h; 2) piperidine, DMF, rt, 30 min; b: 1) **8**, HOBt, DIC, NEt_3_, DMF, rt, 72 h; 2) TFA/H_2_O/triisopropysilane (95:2.5:2.5), rt, 3 h.

Thiourea **3** was prepared as described before [15], starting with the Boc-protection of tris(2-aminoethyl)amine (**1**) followed by the stepwise reaction of the free NH_2_ group of product **2** with thiocarbonyldiimidazole and methyl 3,4-diaminobenzoate (Scheme 2). The cyclisation to form the first benzimidazole unit was then achieved by reacting thiourea **3** with Mukaiyama’s reagent in the presence of NEt_3_ at rt yielding 88% of **4**. Although the yield is slightly lower compared to the cyclisation with HgO (97% yield), the mild and metal-free reaction conditions are a big advantage of this new method. For the subsequent two conversions, again the known procedures were used. Thiourea **6** was prepared by deprotection of **4** with SOCl_2_ in MeOH and addition of 2-nitrophenylisothiocyanate followed by reduction of the nitro groups with H_2_. In the last step of the synthesis, again Mukaiyama’s reagent was used for the formation of the two benzimidazole moieties. Stirring at rt for 22 h in the presence of NEt_3_ yielded 56% of tris(2-aminobenzimidazole) **7** after HPLC purification. Ester hydrolysis with aq HCl led to the free carboxylic acid **8**, which after removal of the solvent was used for conjugation to PNA without further purification (Scheme 3).

Conjugation of tris(2-aminobenzimidazole) **8** was performed with fully protected PNA-oligomers still bound to the solid support. To increase the solubility of the final product, 10mers were attached to one, 15mers to two lysine units placed at the C-terminus. For conjugation, the Fmoc-protected 6-aminohexanoic acid **9** was bound to the PNA at the terminal amino group as a linker. After deprotection of the linker’s amino function, tris(2-aminobenzimidazole) was attached. In both steps, DIC and HOBt were used as reagents. Coupling of the cleaver proceeded in high yields with no unconjugated PNA detectable in the MALDI mass spectra of the crude products. All conjugates were purified by RP-HPLC and isolated as trifluoroacetate salts. Starting from 20–30 mg of Rink amide MBHA resin (0.66 mmol/g) even manual synthesis routinely yielded milligram amounts of the purified final product. This compares favorably to the yield of DNA conjugates we have studied earlier [16].

**Figure 1:**
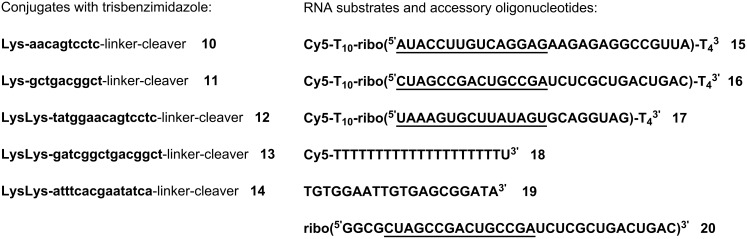
Sequences of PNA conjugates **10–14** and oligonucleotides **15–20**. Lysines are attached to the *C*-terminus, linker and cleaver to the *N*-terminus. The parts with base complementarity to 15mer PNAs, i.e., the binding sites, are underlined. When 10mer PNAs bind to substrates **15** and **16**, the first five ribonucleotides at the 5’ end will remain single stranded.

Cleavage experiments were run with Cy5-labeled RNA, the fluorescent dye permitting the detection and quantification of fragments by gel electrophoresis in a DNA sequencer (Figure 1). Substrates **15** and **16** have been used for comparison with our previous studies [16]. The third substrate **17** has the sequence of miRNA 20a, a member of the oncogenic miRNA 17–92 cluster [19] that is a promising target for site-specific RNA cleavers. All RNA parts of substrates **15–17** are embedded into a stretch of nonreacting deoxythymidine residues to improve the separation and resolution of substrate and fragment peaks in the sequencer.

**Figure 2:**
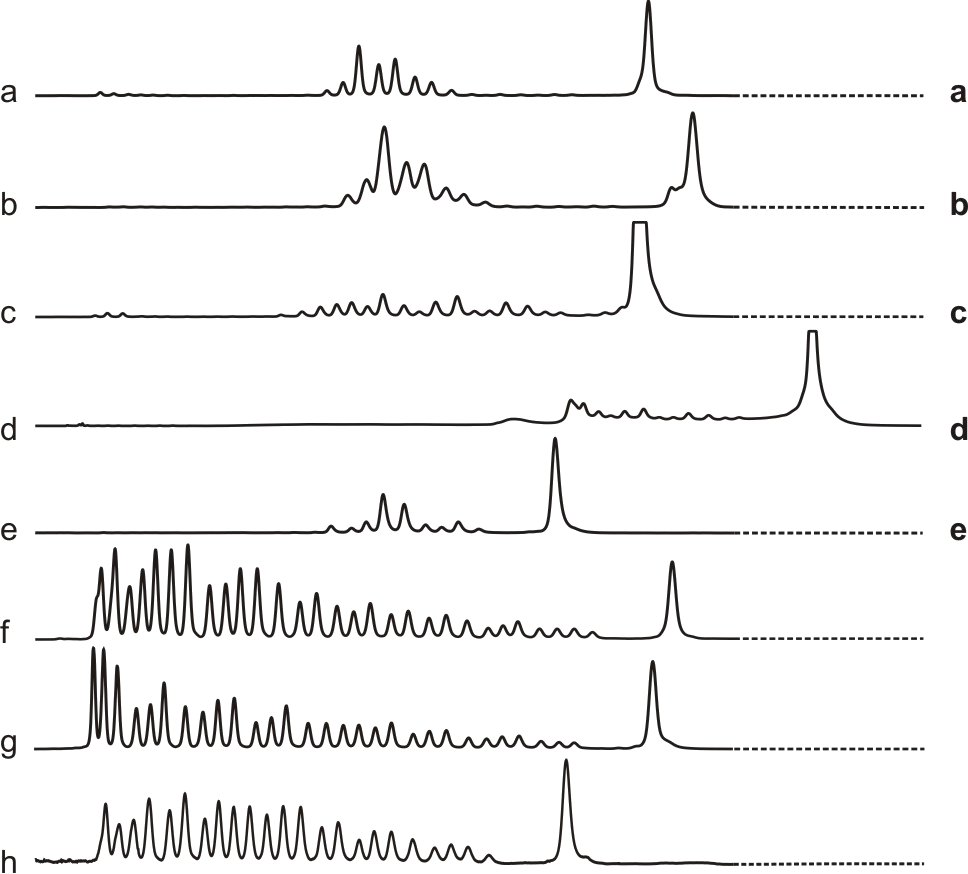
Cleavage of RNA by their corresponding PNA conjugates (150 nM substrate, 750 nM conjugate, 50 mM Tris-HCl, pH 8, 37 °C, 20 h). Lane a: conjugate **10** and substrate **15**. Lane b: conjugate **12** and substrate **15**. Lane c: conjugate **11** and substrate **16**. Lane d: conjugate **13** and substrate **16**. Lane e: conjugate **14** and substrate **17**. Lanes f, g and h: hydrolysis patterns of **15**, **16** and **17** (Na_2_CO_3_).

Incubation of RNA **15** with conjugate **12**, which has the same 15mer sequence as a previously tested DNA conjugate [16], led to 8 dye labeled fragments, all located in the non-hybridized part of the substrate. Most prominent is cleavage after G(18) (Figure 2, lane b; note the slight shift and duplication of the substrate peak compared to the hydrolysis ladder shown in lane f). The product distribution is considerably broader compared to degradation of RNA **15** by the corresponding DNA conjugate. Similar results were obtained by reaction of substrate **17** with 15mer-conjugate **14** (lane e). Here, A(18) and G(19) are the main cleaving sites. Unexpectedly, no distinct cleavage pattern could be observed with the 15mer-conjugate **13** and its cognate RNA **16**. Instead, the electropherogram showed a shift of several signals including the substrate peak (lane d, compare to hydrolysis ladder of **16** in lane g), making it difficult to assign the signals to individual cleavage fragments. However, the position of the signals relative to the substrate peak suggests that cleavage occurred only in the non-hybridized part of the RNA. We explain this effect by incomplete PNA–RNA strand separation during electrophoresis due to stronger hybridization resulting from the G/C-rich sequence of the conjugate (10 G/C-base pairs in contrast to 7 and 4 for conjugates **12** and **14**, respectively). The fact that incubation of RNA **15** with PNA **12** also leads to a slight, but much weaker shift of signals (lane b), supports this assumption. The first four nucleotides in the single stranded part of RNA **16** where cleavage takes place have only pyrimidine bases, while RNA **15** and **17** have mainly purine bases in the corresponding regions. If the tris(2-aminobenzimidazole) cleaver interacts with the purine bases (e.g., by stacking) it is not impossible that this could also be related to the apparent lower rate and lower selectivity in cleavage of RNA **16** with both **11** and **13**. Consistent with that idea, we previously had observed similar effects when RNAs **15** and **16** were cleaved by DNA-catalyst conjugates analogous to **12** and **13** [16].

As it is known that PNA binds more tightly to RNA than does DNA [5], 10mer-conjugates **10** and **11** complementary to substrates **15** and **16** were also tested in cleavage experiments. Compared to the 15mers, the first 5 monomers were omitted in order to keep the cleavage site unchanged. Results for the incubation of **15** with **10** were as expected (lane a). In contrast to incubation of substrate **16** with 15mer-conjugate **13**, no signal shift is seen with the 10mer-conjugate **11**. However, the cleavage occurs in a nonspecific way in nearly all positions not protected by hybridization with PNA (lane c). Though site specificity is low in this case, conjugate **13** is nevertheless substrate specific and does not degrade RNAs **15** or **17** under identical conditions (Supporting Information File 1). The relatively low site specificity, as discussed above, may be related to the pyrimidine rich sequence of RNA **16**.

To examine whether PNA conjugates **10**–**14** would form molecular aggregates with noncognate oligonucleotides, the diffusion time of Cy5-labeled DNA **18** (19 nM, diluted with 131 nM unlabeled DNA **19**) in absence and presence of different PNA conjugates (750 nM) was studied by fluorescence correlation spectroscopy (FCS) [15,20]. No significant change in diffusion time was observed upon addition of 10mer conjugate **10** to noncognate DNAs **18**/**19**, indicating that no aggregates were formed. In contrast, addition of 10mer conjugate **11** or of 15mer conjugates **12**, **13**, and **14** to DNAs **18**/**19** led to considerable changes in diffusion times, which was most evident for PNAs **11** and **13**. This clearly shows that molecular aggregates of oligonucleotides and PNA conjugates are formed in all of these cases [21]. Although such effects were absent in FCS experiments with analogous DNA conjugates [16], this result is not surprising as PNA, with its uncharged backbone, is more likely to form aggregates. In the presence of 10% of DMSO, no change in diffusion times was observed for conjugates **12** and **14**. Conjugates **11** and **13** still caused an effect but less pronounced than in the absence of DMSO, indicating that DMSO prevents aggregation partially. We also investigated RNA **16** by FCS (19 nM **16**, diluted with 131 nM of the unlabeled analog **20**). Upon addition of 10mer PNA **11**, a large increase of diffusion times was observed that can only be explained by aggregation.

**Figure 3:**
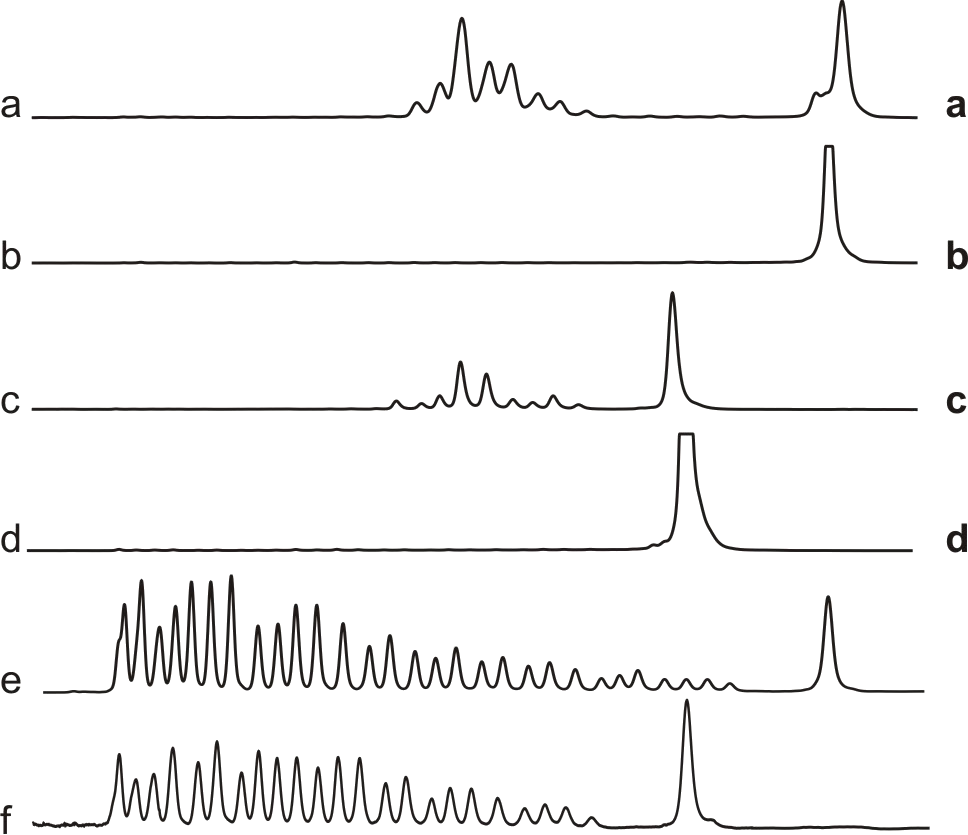
Substrate specificity of conjugates **12** and **14** (150 nM substrate, 750 nM conjugate, 50 mM Tris-HCl, pH 8, 37 °C, 20 h). Lane a: conjugate **12** and substrate **15**. Lane b: conjugate **14** and substrate **15**. Lane c: conjugate **14** and substrate **17**. Lane d: conjugate **12** and substrate **17**. Lanes e and f: hydrolysis patterns of **15** and **17** (Na_2_CO_3_).

Aggregation of PNA conjugates with complementary RNA but even with noncognate oligonucleotides [21] might cause a loss of sequence specificity. It was of critical importance, therefore, to conduct cross reaction experiments of PNA conjugates **10**–**14** in all possible combinations with substrates **15**–**17**. Exemplarily, Figure 3 shows tests of conjugates **12** and **14** with RNAs **15** and **17**. Consistent data was obtained for all other possible combinations of conjugates and substrates (Supporting Information File 1), which proves that cleavage in the examined concentration range fully depends on Watson–Crick base pairing.

**Figure 4:**
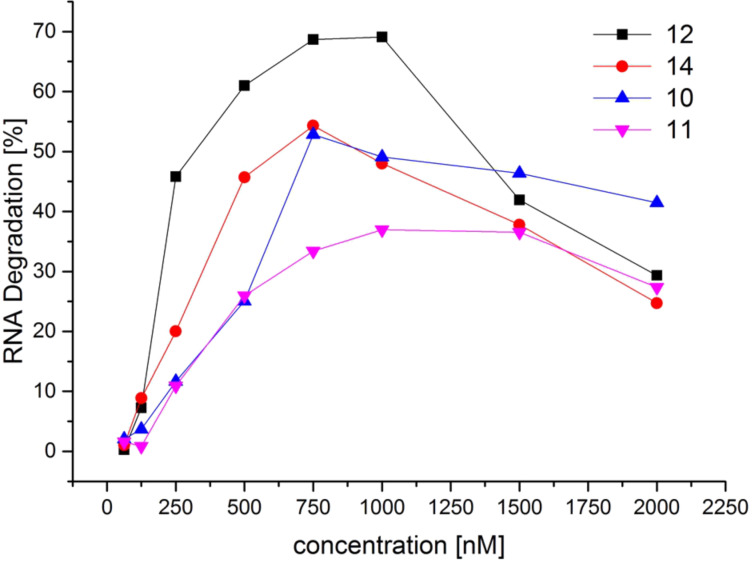
Cleavage of RNA substrates **15**, **16**, and **17** by their matching conjugates as a function of conjugate concentration (150 nM substrate, 62.5–2000 nM conjugate, 50 mM Tris-HCl, pH 8, 37 °C, 20 h). Data points are connected by lines for the sake of clarity.

Cleavage experiments with varying amounts of conjugates (Figure 4) show that – at concentrations below 1.5 µM – RNA cleavage by PNAs **10**–**12** and **14** obeys saturation kinetics. Full activity is reached at concentrations of 500–750 nM. In comparison to the shortened analog **10**, 15mer conjugate **12** shows higher cleavage yields of RNA **15** at concentrations from 62.5 to 1000 nM. Larger amounts of PNA, however, decrease the effectiveness of RNA degradation. This drop is quite considerable for the 15mer conjugates, whereas it is less significant for the 10mer analogs. The decreasing cleavage yields at high PNA concentrations are likely to result from increased aggregation, which might prevent the cleaver from interacting with the RNA backbone.

**Figure 5:**
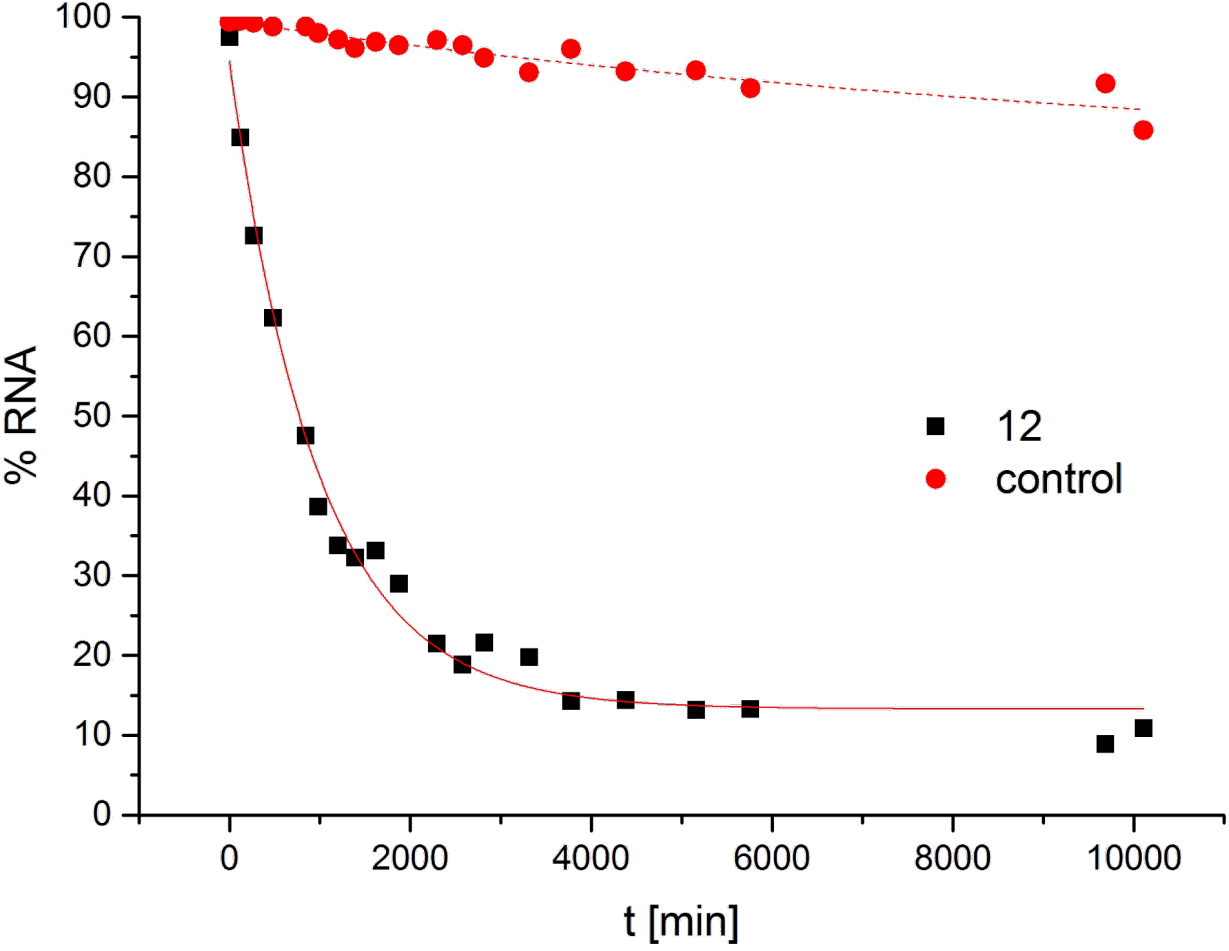
Cleavage kinetics of **15** in the presence and absence of conjugate **12**. Conditions: 150 nM substrate, 0 or 750 nM conjugate, 50 mM Tris-HCl, pH 8, 100 mM NaCl, 37 °C. The solid curves are calculated assuming first order kinetics.

For the reaction of substrate **15** with conjugate **12**, cleavage kinetics were studied in detail. Figure 5 shows RNA decay under saturation conditions as a function of time. While in the absence of a cleaver the substrate proved to be quite stable for several days, it was cleaved significantly by conjugate **12** within a few hours. Almost complete degradation was achieved after 60 h. As seen before [16], a small percentage of RNA remained intact even after one week. This could be due to structural imperfections in the synthetic substrate RNA preventing the PNA conjugate from hybridizing with its target molecule. The best fit of data to a first order rate law is shown as a solid curve: *k_1_* = 0.062 h^−1^, *t_1/2_* = 11.2 h. This number comes close to the half-life of **15** when cleaved by the analogous DNA conjugate (*t_1/2_* = 12.4 h) [16].

## Conclusion

We have shown that PNA conjugates of tris(2-aminobenzimidazole) are potent hydrolytic cleavers of RNA, exhibiting similar efficiency and substrate specificity as the corresponding DNA conjugates tested previously. Our optimized synthesis of tris(2-aminobenzimidazole) provides a new way to obtain the catalyst avoiding mercury salts with all their disadvantages (toxicity, harsh reaction conditions, variable yields). Conjugation to PNA oligomers can be readily achieved in high yields, providing an easy way to synthesize sequence specific metal-free artificial nucleases for a wide range of RNA substrates. Due to the tendency of PNA conjugates to form aggregates, a phenomenon not seen with their DNA analogs, optimization of the PNA oligomer length is necessary, especially for strands rich in G/C base pairs. Reduced activity of long PNA conjugates caused by aggregation can be a minor disadvantage in comparison to DNA conjugates. However, the higher affinity of PNA towards RNA allows using shorter PNA oligomers. In addition, the stability of PNA against biodegradation is another advantage especially with respect to possible in cell applications.

## Supporting Information

File 1 Experimental procedures and characterization data.
