# Supplementary material for: Sequence-specific RNA cleavage by PNA conjugates of the metal-free artificial ribonuclease tris(2-aminobenzimidazole)
Source: Beilstein J Org Chem. 2015 Apr 16;11:493–8. doi: 10.3762/bjoc.11.55 (PMC4419560; doi:10.3762/bjoc.11.55)

Supporting Information

for

Sequence-specific RNA cleavage by PNA conjugates of the metal-free artificial ribonuclease tris(2-aminobenzimidazole)

Friederike Danneberg[1], Alice Ghidini[2], Plamena Dogandzhiyski[1], Elisabeth Kalden[1], Roger Strömberg[2] and Michael W. Göbel*[1]

Address: [1]Institute for Organic Chemistry and Chemical Biology, Goethe Universität Frankfurt, Max-von-Laue-Straße 7, D-60438 Frankfurt am Main, Germany and [2]Karolinska Institutet, Department of Biosciences and Nutrition, Novum, SE-141 83 Huddinge, Sweden

Email: Michael Göbel - m.goebel@chemie.uni-frankfurt.de

*Corresponding author

Experimental procedures and characterization data

Table of contents:

General.. S2
Synthesis of tris(2-aminobenzimidazole) **7**... S2
Synthesis of PNA conjugates..S7
References..S10
Substrate specificity.. S11
NMR spectra... S12

General

All chemicals were reagent grade and used as purchased. All of the reactions except ester hydrolysis were performed under an argon atmosphere. Reactions were monitored by TLC using Merck TLC silica gel 60 F-254 aluminium sheets. Compounds were visualized by UV light (254 and 366 nm) or by staining with a solution of ninhydrin in ethanol. Column chromatography was carried out on silica gel 60 (0.04–0.063 mm).

Melting points were recorded on a Kofler system. ^{1}H and ^{13}C NMR spectra were recorded on a BRUKER DPX 250, a Bruker AM 300 or a BRUKER AV 500 spectrometer. Chemical shifts are expressed in parts per million (ppm) relative to the partially nondeuterated solvent signal DMSO-d_5 (δ_H = 2.50, δ_C = 39.43) or $CHCl_3$ (δ_H = 7.26, δ_C = 77.00) as an internal reference. IR spectra were recorded with a Perkin Elmer 1600 Series FTIR spectrometer (using a KBr pellet for solids and NaCl plates for liquids) or a Perkin Elmer Spectrum Two FTIR spectrometer equipped with a Perkin UATR Two unit. ESI mass spectrometry was performed on a Fisons VG Platform II spectrometer or on a nano-ESI spectrometer (Perseptive Biosystems, Mariner). High-resolution mass spectra (HRMS) were obtained with a Thermo Scientific MALDI LTQ Orbitrap. Elemental analysis was carried out with a Vario MICRO cube apparatus.

Preparative HPLC of tris(2-aminobenzimidazole) **7** was performed on a Waters pump 590 with a Beckman 163 variable wavelength detector. Analytical HPLC was performed on a JASCO PU-980 with a UV-975 UV/vis detector and a Varian 385-LC detector.

Synthesis of the tris(2-aminobenzimidazole) derivative 7

Bis-[2-(*tert*-butoxycarbonylamino)ethyl]-(2-aminoethyl)amine (2). In a similar manner as described in [1], a solution of tris(2-aminoethyl)amine **1** (1.42 mL, 9.52 mmol) in 50 mL of THF (abs.) was cooled to 0° C. A solution of Boc-ON (4.95 g, 20.1 mmol) in 50 mL of THF (abs.) was added dropwise during 2.5 h. The yellow solution was stirred for 46 h at rt. After removal of the solvent under reduced pressure, the remaining oil was dissolved in 50 mL of ethyl acetate and washed twice with 0.5 M NaOH. The combined aqueous phases were diluted with brine and

reextracted with ethyl acetate thrice. The combined organic phases were dried over $MgSO_4$ and the solvent was evaporated at reduced pressure. After column chromatography of the crude product (DCM/MeOH 5:1 + 1% NEt_3), 1.49 g of the product compound were obtained as a yellow oil (45%). R_f = 0.27 (DCM/MeOH 5:1, 1% NEt_3). ^{1}H-NMR (250 MHz, DMSO-d_6): δ = 6.75 – 6.71 (m, 2 H, NH), 2.98 – 2.91 (m, 4 H, CH_2), 2.54 (t, 2 H, J = 5.8 Hz, CH_2, overlaps with DMSO), 2.44 – 2.39 (m, 6 H, CH_2), 1.37 (s, 18 H, CH_3). ^{13}C-NMR (62.9 MHz, DMSO-d_6): δ = 155.6, 77.3, 56.6, 53.9, 38.3, 28.1. IR (KBr): 3355 (m), 2977 (m), 2933 (m), 2819 (w), 1700 (s), 1522 (s), 1458 (m), 1391 (m), 1365 (s), 1275 (m), 1251 (s), 1174 (s), 1068 (w), 970 (w), 864 (w), 781 (w). MS (ESI$^+$): m/z (%) = 347.2 (100) $[M+H]^+$, calcd for $C_{16}H_{34}N_4O_4$: 346.26. HRMS (MALDI) m/z = 347.26612 $[M+H]^+$, calcd for $C_{16}H_{34}N_4O_4+H^+$: 347.26528.

4-Amino-3-(3-{2-[bis-(2-{*tert*-butoxycarbonylamino}ethyl)amino]ethyl}thio-ureido)benzoic acid methyl ester (3). In a similar manner as described in [1], a solution of amine **2** (1.46 g, 4.20 mmol) in 15 mL MeCN (abs.) was added dropwise at 0 °C to a solution of 1,1‘-thiocarbonyl diimidazole (1.12 g, 6.30 mmol) and imidazole (94 mg, 1.39 mmol) in 25 mL MeCN (abs.). The reaction mixture was stirred for 1 h at rt. Methyl 3,4-diaminobenzoic acid (1.05 g, 6.30 mmol) was dissolved in 10 mL MeCN (abs.) and added dropwise to the reaction mixture. The solution was stirred for 4 h at 50 °C and at rt overnight. The solvent was evaporated at low pressure and the residue purified by column chromatography (*n*-hexane/ethyl acetate 1:1 → ethyl acetate), yielding 1.83 g (79%) of the product compound as a yellow foam. R_f = 0.48 (ethyl acetate). ^{1}H-NMR (250 MHz, DMSO-d_6): δ = 8.85 (br s, 1 H, NH-Aryl), 7.60 – 7.55 (m, 2 H, Aryl-H), 7.33 (br s, 1 H, NHC=S), 6.75 (d, 1 H, J = 9.0 Hz, Aryl-H), 6.69 – 6.61 (m, 2 H, NHC=O), 5.68 (br s, 2 H, NH_2), 3.74 (s, 3 H, OCH_3), 3.49 – 3.41 (m, 2 H, CH_2NC=S), 2.97 – 2.90 (m, 4 H, CH_2NC=O), 2.60 – 2.54 (m, 2 H, CH_2), 2.48 – 2.44 (m, 4 H, CH_2, overlaps with DMSO), 1.37 (s, 18 H, CH_3). ^{13}C-NMR (125.8 MHz, DMSO-d_6): δ = 181.2, 165.9, 155.6, 149.2, 130.3, 128.9, 122.0, 116.3, 114.4, 77.5, 53.5, 52.3, 51.3, 42.1, 38.2, 28.2. IR (KBr): 3321 (m), 2977 (m), 1689 (s), 1623 (m), 1513 (s), 1436 (m), 1391 (m), 1365 (m), 1287 (m), 1246 (s), 1161 (s), 1102 (m), 974 (m), 858 (w), 768 (m), 619 (w). MS (ESI$^+$): m/z (%) = 556.5 (100) $[M+H]^+$, calcd for $C_{25}H_{42}N_6O_6S$: 554.29. HRMS (MALDI) m/z = 555.29558 $[M+H]^+$, calcd for $C_{25}H_{42}N_6O_6S+H^+$: 555.29593.

2-{2-[Bis-(2-{*tert*-butoxycarbonylamino}ethyl)amino]ethylamino}-1*H*-benzimid-azole-5-carboxylic acid methyl ester (4) [1]. To the solution of thiourea **3** (1.67 g, 3.01 mmol) and NEt_3 (0.93 mL, 6.62 mmol) in 11 mL dry DMF, 2-chloro-1-methylpyridinium iodide (Mukaiyama's reagent, 922 mg, 3.61 mmol) was added in several portions. The yellow solution was stirred for 20 h at rt. The solution was filtered and the solvent removed in vacuum. The residue was purified by chromatography on silica gel (ethyl acetate/methanol 19:1), yielding 1.39 g (88%) of the product compound as a yellow foam. R_f = 0.62 (ethyl acetate/MeOH 5:1). ^{1}H-NMR (250 MHz, DMSO-d_6): δ = 10.92 (br s, 1 H, NH), 7.72 (d, 1 H, *J* = 1.3 Hz, Aryl-H), 7.58 (br. d, 1 H, *J* = 8.3 Hz, Aryl-H), 7.17 (d, 1 H, *J* = 8.3 Hz, Aryl-H), 6.85 (br s, 1 H, NH-Aryl), 6.77 – 6.73 (m, 2 H, NHC=O), 3.80 (s, 3 H, OCH_3), 3.37 – 3.30 (m, 2 H, CH_2N-Aryl, overlaps H_2O), 3.02 – 2.94 (m, 4 H, CH_2NC=O), 2.67 – 2.62 (m, 2 H, CH_2), 2.55 (m, 4 H, CH_2, overlaps with DMSO), 1.35 (s, 18 H, CH_3). ^{13}C-NMR (62.9 MHz, DMSO-d_6): δ = 167.0, 157.3, 155.5, 121.5, 77.4, 53.8, 53.6, 51.4, 38.3, 28.1. IR (KBr): 3365 (m), 2976 (m), 2933 (m), 2867 (w), 1686 (s), 1636 (m), 1601 (m), 1578 (m), 1522 (m), 1511 (m), 1461 (m), 1438 (m), 1389 (w), 1366 (m), 1291 (s), 1250 (m), 1168 (m), 1089 (w), 776 (w). MS (ESI$^+$): m/z (%) = 522.0 (100) $[M+H]^+$, calcd for $C_{25}H_{40}N_6O_6$: 520.3. HRMS (MALDI) m/z = 521.30784 $[M+H]^+$, calcd for $C_{25}H_{40}N_6O_6+H^+$: 521.30821.

2-[2-(Bis-{2-[3-(2-nitrophenyl)thioureido]ethyl}amino)ethylamino]-1*H*-benzimid-azole-5-carboxylic acid methyl ester (5). This compound was prepared in a similar manner as described in [1]: To a solution of compound **4** (639 mg, 0.99 mmol) in 12 mL dry MeOH, $SOCl_2$ (0.71 mL, 9.85 mmol) was added dropwise at 0 °C. The yellow solution was stirred for 3 h at rt. After evaporation of the solvent, the residue was dissolved in a solution of 4 mL of NEt_3 and 8 mL of dry ethanol and added dropwise to the solution of 2-nitrophenylisothiocyanate (391 mg, 2.17 mmol) and 4 mL of NEt_3 in 12 mL of dry EtOH at 0 °C. The reaction mixture was stirred at rt overnight. Evaporation of the solvent and column chromatography (ethyl acetate → ethyl acetate/MeOH 5:1) yielded 608 mg (90%) of thiourea **5** as a yellow foam. R_f = 0.49 (DCM/MeOH 5:1 + 1% NEt_3). ^{1}H-NMR (250 MHz, DMSO-d_6): δ = 10.99 (br s, 1 H, NH), 9.79 (s, 2H, C=S-NH-Aryl), 8.44 – 8.40 (m, 2 H, NHC=S), 7.97 (d, 2 H, *J* = 8.3 Hz, Aryl-H), 7.77 – 7.57 (m, 6 H, Aryl-H), 7.36 (t, 2 H, *J* = 7.5 Hz, Aryl-H), 7.18 (d, 1 H, J = 8.0 Hz, Aryl-H), 6.99 (br s, 1 H, NH-Aryl), 3.80 (s, 3 H, OCH_3), 3.66 – 3.57

(m, 4 H, CH_2), 3.50 – 3.43 (m, 2 H, CH_2), 2.85 – 2.77 (m, 6 H, CH_2). ^{13}C-NMR (62.9 MHz, DMSO-d_6): δ = 181.3, 166.8, 156.7, 143.2, 133.5, 133.3, 128.8, 125.4, 124.5, 121.8, 79.0, 53.1, 52.0, 51.4, 42.0. IR (KBr): 3295 (m), 3034 (w), 2945 (w), 2845 (w), 1702 (w), 1686 (w), 1636 (m), 1606 (m), 1578 (m), 1508 (s), 1458 (m), 1436 (m), 1340 (m), 1285 (s), 1202 (m), 1158 (w), 1088 (w), 747 (w), 724 (w). MS (ESI^-): m/z (%) = 679.7 (91) $[M-H]^-$, 715.9 (100) $[M+Cl]^-$, 807.9 (77) $[M+I]^-$, calcd for $C_{29}H_{32}N_{10}O_6S_2$: 680.19. HRMS (MALDI) m/z = 681.20134 $[M+H]^+$, calcd for $C_{29}H_{32}N_{10}O_6S_2+H^+$: 681.20205.

2-[2-(Bis-{2-[3-(2-aminophenyl)thioureido]ethyl}amino)ethylamino]-1*H*-benzimidazole-5-carboxylic acid methyl ester (6). This compound was prepared in a similar manner as described in [1]: Nitro compound **5** (735 mg, 1.08 mmol) was dissolved in 40 mL of dry MeOH saturated with ammonia. After addition of palladium on activated charcoal (160 mg) the reaction mixture was stirred at 60 °C in an autoclave at 45 bar hydrogen pressure for 5 h and at 40 bar hydrogen pressure and rt overnight. The mixture was filtrated over Celite and evaporated to dryness. Column chromatography (DCM/MeOH 95:5) yielded 250 mg (37%) of the diamine **6** as an off-white foam. R_f = 0.43 (acetic acid/MeOH 9:1). ^{1}H-NMR (250 MHz, DMSO-d_6): δ = 10.94 (br s, 1 H, NH), 8.94 (s, 2H, NH), 7.72 (d, 1H, J = 1.6 Hz, Aryl-H), 7.58 (dd, 1H, J = 8.2 Hz, J = 1.3 Hz, Aryl-H), 7.26 – 7.13 (m, 3 H, Aryl-H und NH), 7.00 – 6.91 (m, 4 H, Aryl-H), 6.81 (br s, 1 H, NH), 6.77 – 6.73 (m, 2H, Aryl-H), 6.55 (dd, 2 H, J = 7.5 Hz, J =1.2 Hz), 4.84 (br s, 4 H, NH_2), 3.80 (s, 3 H, OCH_3), 3.54 – 3.46 (m, 4 H, CH_2), 3.37 – 3.31 (m, 2 H, CH_2, overlaps with H_2O), 2.73 – 2.64 (m, 6 H, CH_2). IR (neat): 3210 (m), 2927 (m), 1695 (m), 1599 (m), 1575 (m), 1532 (s), 1497 (s), 1456 (m), 1435 (m), 1285 (s), 1233 (m), 1156 (m), 1088 (m), 745 (m), 614 (w). MS (ESI^+): m/z (%) = 622.0 (100) $[M+H]^+$, calcd for $C_{29}H_{36}N_{10}O_2S_2$: 620.25. HRMS (MALDI) m/z = 621.25279 $[M+H]^+$, calcd for $C_{29}H_{36}N_{10}O_2S_2+H^+$: 621.25369.

2-(2-{Bis-[2-(1*H*-benzimidazol-2-ylamino)ethyl]amino}ethylamino)-1*H*-benzimidazole-5-carboxylic acid methyl ester (7) [1]. To the solution of thiourea **6** (240 mg, 0.39 mmol) and NEt_3 (0.24 mL, 1.70 mmol) in 3.7 mL dry DMF, Mukaiyama's reagent (237 mg, 0.93 mmol) was added in several portions. The yellow solution was stirred for 22 h at rt. The solution was filtered and the solvent removed in vacuum. The residue was purified first by chromatography on silica gel (DCM/MeOH 9:1 + 1%

NEt_3), then by HPLC, yielding 251 mg (56%) of the product compound as an off-white solid. Mp: 179 – 181 °C. ^{1}H-NMR (250 MHz, DMSO-d_6): δ = 9.22 (br s, 1 H, NH), 9.06 – 8.98 (m, 2 H, NH), 7.84 – 7.82 (m, 2 H, Aryl-H), 7.42 (d, 1 H, *J* = 8.8 Hz, Aryl-H), 7.36 – 7.32 (m, 4 H, Aryl-H), 7.23 – 7.19 (m, 4 H, Aryl-H), 3.86 (s, 3 H, OCH_3), 3.65 – 3.52 (m, 6 H, CH_2), 3.09 – 2.97 (m, 6 H, CH_2). IR (neat): 2971 (m), 2904 (m), 2755 (m), 2656 (m), 1658 (s), 1619 (s), 1476 (m), 1436 (m), 1389 (m), 1289 (m), 1232 (m), 1205 (m), 1139 (m), 1087 (m), 1015 (w), 982 (w), 897 (w), 738 (m), 685 (m), 619 (m). Elemental analysis (%) for $C_{29}H_{32}N_{10}O_2 \cdot 6$ TFA · H_2O (1254.79): calcd: C, 39.24; H, 3.21; N, 11.16; found: C, 39.18; H, 3.28; N, 11.34. MS (ESI$^+$): m/z (%) = 553.4 (100) $[M+H]^+$, calcd for $C_{29}H_{32}N_{10}O_2$: 552.3. HPLC conditions: semi-preparative: ReproSil-Pur C18-AQ, 250 x 20, 10 µm, 0.1 % TFA/MeCN (72:28), 9.5 mL/min, 254 nm; analytical: ReproSil-Pur C18-AQ, 125 x 4.6, 5 µm, 0.1 % TFA/MeCN (70:30), 0.8 mL/min, 254 nm, t_R = 3.99 min.

2-(2-{Bis-[2-(1*H*-benzimidazol-2-ylamino)ethyl]amino}ethylamino)-1*H*-benzimid-azole-5-carboxylic acid (8). This compound was prepared in a similar manner as described in [1]: 239 mg (0.21 mmol) of the TFA-salt of **7** were converted to the HCl-salt using DOWEX ion exchange resin (Cl^--form). This was then dissolved in 7.5 mL H_2O (dest.) and 7.5 mL of conc. HCl were added. The solution was refluxed for 2 h and the solvent was removed in vacuo, yielding the desired free acid quantitatively as a colourless solid that was used for the synthesis of PNA conjugates without further purification. MS (ESI$^-$): m/z (%) = 573.3 (100) $[M+Cl]^-$, calcd for $C_{28}H_{30}N_{10}O_2$: 538.3.

6-{[(9*H*-Fluoren-9-ylmethoxy)carbonyl]amino}hexanoic acid (9). To a solution of 389 mg (2.96 mmol) 6-aminohexanoic acid in 20 mL acetone/water (1:1), 1.00 g (2.96 mmol) FmocOSuc and 250 mg (2.96 mmol) $NaHCO_3$ were added. The suspension was stirred at rt for 3 h. Acetone was removed at reduced pressure and 20 mL of DCM were added to the residue. The precipitated solid was removed by filtration. The organic phase was washed with 10 mL of 0.1 M HCl and 10 mL of water. The solution was dried over Na_2SO_4, the solvent removed at reduced pressure and the residue was dried *in vacuo*, yielding 747 mg (71%) of a colourless solid. R_f = 0.61 (DCM/MeOH 9:1). Mp: 116 – 117 °C (Lit.: 114 °C [2]). ^{1}H-NMR (250 MHz, $CDCl_3$): δ = 7.77 (d, 2 H, *J* = 7.6 Hz, Aryl-H), 7.59 (d, 2 H, *J* = 7.5 Hz, Aryl-H), 7.43 – 7.37 (m, 2 H, Aryl-H), 7.31 (dt, 2 H, *J* = 1.2 Hz, *J* = 7.4 Hz, Aryl-H), 4.82 – 4.72 (m, 1

H, NH), 4.41 (d, 2 H, J = 6.9 Hz, CH_2(Fmoc)), 4.22 (t, 1 H, J = 6.7 Hz, CH(Fmoc)), 3.24 – 3.16 (m, 2 H, H-C(6)), 2.36 (t, 2 H, J = 7.3 Hz, H-C(2)), 1.72 – 1.32 (m, 6 H, H-C(3), H-C(4), H-C(5)). ^{13}C-NMR (75.5 MHz, $CDCl_3$): δ = 178.8, 156.4, 144.0, 141.3, 127.6, 127.0, 125.0, 119.9, 66.5, 47.3, 40.8, 33.7, 29.6, 26.1, 24.2. IR (neat): 3339 (m), 2952 (w), 1688 (s), 1530 (s), 1478 (w), 1462 (m), 1450 (m), 1415 (w), 1330 (m), 1292 (m), 1270 (m), 1253 (m), 1238 (m), 1197 (m), 1172 (w), 1131 (m), 1102 (m), 996 (m), 934 (m), 759 (m), 736 (s), 577 (m), 528 (m). (ESI$^+$): m/z (%) = 354.1 (100) $[M+H]^+$, calcd for $C_{21}H_{23}NO_4$ 353.2.

Synthesis of PNA conjugates

Synthesis of PNA 10mers (The method described here closely resembles a method we published earlier [3] for the synthesis of tripeptides unrelated to PNA). PNA 10mers were assembled manually on a solid support (Rink Amide MBHA resin purchased from Novabiochem, now incorporated into Merck Millipore, Darmstadt, Germany) by using 2 mL polyethylene syringe reactors that were equipped with a fritted disc. Peptide nucleic acid monomers were purchased from Link Technologies (Bellshill, UK). Fmoc-$^{\alpha}$N-Lys($^{\varepsilon}$N-Boc)OH, HOBt and DIC were from Sigma-Aldrich. The 10mers were synthesized using the following procedure: After swelling the resin (100 mg) with DCM for 1.5 h, the solvent was substituted by NMP. To release the amine the Fmoc-protected resin was treated with piperidine (20% in DMF) three times (15 min, 10 min, 5 min). Thereafter, the resin was washed with NMP 5 times. For coupling, the Fmoc-amino acid or the Fmoc-PNA monomer (3 equiv) was dissolved in 1 mL of NMP together with HOBt (3 equiv) and DIC (3 equiv). After shaking for 5 min it was added to the resin. The mixture was shaken at rt until the Kaiser test showed complete coupling. The resin was repeatedly washed with NMP and the Fmoc group was removed as described before. After washing with NMP for 5 times, subsequent Fmoc-protected monomer was attached using the same procedure as before. For the storage of the fully assembled and still Fmoc-protected PNA 10mer, the resin was washed with DCM for 10 times, dried in vacuo and stored at −20 °C until being used for conjugation of the cleaver. A small sample was treated with 1.5 mL of a 9.5:0.25:0.25 mixture of TFA/H_2O/TIPS twice (3 h and 30 min). The combined cleaving solutions were evaporated to dryness and the residue was suspended with cold diethyl ether three times and spun down with a centrifuge

(6000 rpm). The pellet was dried in an air flow, redissolved in H_2O and evaporated to dryness. A mass spectrum (MALDI) was recorded to confirm the complete assembly of the respective 10mer. MALDI was performed on a Voyager-DE STR spectrometer (Perseptive Biosystems).

Synthesis of PNA 15mers. PNA 15mers were assembled automatically on a solid support (Rink Amide resin), using the manufacturer’s protocol for the Biotage Initiator+ Alstra microwave peptide synthesizer with 9-fluorenylmethyloxycarbonyl (Fmoc)-chemistry. Fmoc deprotection was carried out with 20% piperidine/NMP at rt and PNA monomer coupling was carried out using ethyl (*E*)-2-cyano-2-(hydroxyimino)acetate (Oxyma) and diisopropyl carbodiimide (DIC) as coupling agents under microwave irradiation for 6 min at 75 ºC.
Peptide nucleic acid monomers were from Link technologies (Bellshill, UK) and Fmoc-$^{\alpha}$N-Lys($^{\varepsilon}$N-Boc)OH was from Iris Biotech GmbH (Marktredwitz, Germany). Rink Amide ChemMatrix resin was purchased from Biotage (Uppsala, Sweden). Oxyma (ethyl cyano(hydroxyimino)acetate) and DIC (*N*,*N*'-diisopropylcarbodiimide) were purchased from Novabiochem (now incorporated into Merck Millipore, Darmstadt, Germany). Solvents and reagents for solid-phase synthesis were synthesis grade from Applied Biosystems (now incorporated into Life Technologies Europe, Stockholm, Sweden) and Iris Biotech GmbH.

Synthesis of PNA conjugates 10–14. 20 mg of the respective solid phase bound and fully protected PNA was swollen in DCM for 30 min, repeatedly washed with DMF and then treated with piperidine (20% in DMF) for three times (15, 10 and 5 min) to liberate the terminal amino group. Afterwards, the resin was washed with DMF 5 times. For the coupling of the linker, Fmoc protected 6-aminohexanoic acid (25 mg, 72 µmol) was dissolved in 360 µL DMF and HOBt (11 mg, 72 mmol) and DIC (11 µL, 72 µmol) were added. The solution was shaken for 5 min and then added to the resin bound PNA. The mixture was shaken at rt overnight until the Kaiser test showed complete coupling. The resin was repeatedly washed with DMF, treated with piperidine (20 % in DMF) for three times (15, 10 and 5 min) and again washed with DMF 5 times. For the coupling of the cleaver, trisaminobenzimidazole **8** (50 mg, 72 µmol) was dissolved in 360 µL DMF and HOBt (11 mg, 72 µmol), NEt_3 (40 µL, 0.29 mmol) and DIC (11 µL, 72 µmol) were added. The solution was shaken for 5 min

and then added to the resin bound PNA. The mixture was shaken at rt for 72 h until the Kaiser test showed almost complete coupling. The resin was washed with DMF, DCM and MeOH 5 times respectively, dried in vacuo and treated with 1.5 mL of a 9.5:0.25:0.25 mixture of TFA/H_2O/TIPS twice (3 h and 30 min). The combined cleaving solutions were evaporated to dryness and the residue was suspended with cold diethyl ether three times and spun down with a centrifuge (6000 rpm). The pellet was dried in an air flow, redissolved in H_2O, evaporated to dryness and purified by HPLC.

HPLC purification of PNA conjugates. PNA conjugates were purified by RP-HPLC. Preparative HPLC of conjugate **10** was performed on a JASCO PU-2080 with a UV-2075 UV–vis detector. Analytical HPLC of conjugate **10** and analytical as well as preparative HPLC of conjugates **11**, **12**, **13**, and **14** was performed on a JASCO PU-980 with a UV-975 UV/vis detector and a Varian 385-LC detector. Columns were heated to 50 °C.

Conjugate 10. MS (MALDI) m/z = 3433.5 $[M+H]^+$, calcd for $C_{146}H_{186}N_{70}O_{32}+H^+$: 3432.5. HPLC conditions: preparative: Phenomenex Jupiter C12 Proteo 90 Å, 250 x 15, 4 µm, linear gradient of 0 - 27 % MeCN in 0.1 % TFA for 20 min, 6.0 mL/min, 254 nm; analytical: Phenomenex Gemini C18, 150 x 4.6, 5 µm, linear gradient of 1 - 50 % MeCN in 0.1 % TFA for 40 min, 1 mL/min, 254 nm, t_R = 14.73 min.

Conjugate 11. MS (MALDI) m/z = 3504.6 $[M+H]^+$, calcd for $C_{147}H_{186}N_{72}O_{34}+H^+$: 3504.5. HPLC conditions: preparative: Phenomenex Gemini C18, 250 x 10, 10 µm, linear gradient of 0 - 35 % MeCN in 0.1 % TFA for 25 min, 4.0 mL/min, 254 nm; analytical: Phenomenex Gemini C18, 150 x 4.6, 5 µm, linear gradient of 1 - 35 % MeCN in 0.1 % TFA for 20 min, 0.8 mL/min, 254 nm, t_R = 17.99 min.

Conjugate 12. MS (MALDI) m/z = 4948.3 $[M+H]^+$, calcd for $C_{207}H_{265}N_{101}O_{49}+H^+$: 4950.1. HPLC conditions: preparative: Phenomenex Gemini C18, 250 x 10, 10 µm, linear gradient of 0 - 50 % MeCN in 0.1 % TFA for 55 min, 4.0 mL/min, 254 nm; analytical: Phenomenex Gemini C18, 150 x 4.6, 5 µm, linear gradient of 1 - 50 % MeCN in 0.1 % TFA for 40 min, 1 mL/min, 254 nm, t_R = 14.99 min.

Conjugate 13. MS (MALDI) m/z = 5006.1 $[M+H]^+$, calcd for $C_{209}H_{266}N_{102}O_{50}+H^+$: 5005.1. HPLC conditions: preparative: Phenomenex Gemini C18, 250 x 10, 10 µm, linear gradient of 0 - 50 % MeCN in 0.1 % TFA for 55 min, 4.0 mL/min, 254 nm; analytical: Phenomenex Gemini C18, 150 x 4.6, 5 µm, linear gradient of 1 - 50 % MeCN in 0.1 % TFA for 40 min, 1 mL/min, 254 nm, t_R = 14.89 min.

Conjugate 14. MS (MALDI) m/z = 4933.2 $[M+H]^+$, calcd for $C_{208}H_{266}N_{100}O_{48}+H^+$: 4933.1. HPLC conditions: preparative: Phenomenex Gemini C18, 250 x 10, 10 µm, linear gradient of 0 - 50 % MeCN in 0.1 % TFA for 60 min, 4.0 mL/min, 254 nm; analytical: Phenomenex Gemini C18, 150 x 4.6, 5 µm, linear gradient of 0 - 50 % MeCN in 0.1 % TFA for 40 min, 1 mL/min, 254 nm, t_R = 22.12 min.

Cleavage and aggregation studies

For experimental details see [1].

References

[1] Scheffer, U.; Strick, A.; Ludwig, V.; Peter, S.; Kalden, E.; Göbel, M. W. *J. Am. Chem. Soc.* **2005**, *127*, 2211–2217.

[2] Fischer-Durand, N.; Salmain, M.; Rudolf, B.; Jugé, L.; Guérineau, V.; Laprévote, O.; Vessières, A.; Jaouen, G. *Macromolecules* **2007**, *40*, 8568–8575.

[3] Ferner, J.; Suhartono, M.; Breitung, S.; Jonker, H. R. A.; Henning, M.; Wöhnert, J.; Göbel, M.; Schwalbe, H. *ChemBioChem* **2009**, *10*, 1490–1494.

Substrate specificity of PNA conjugates

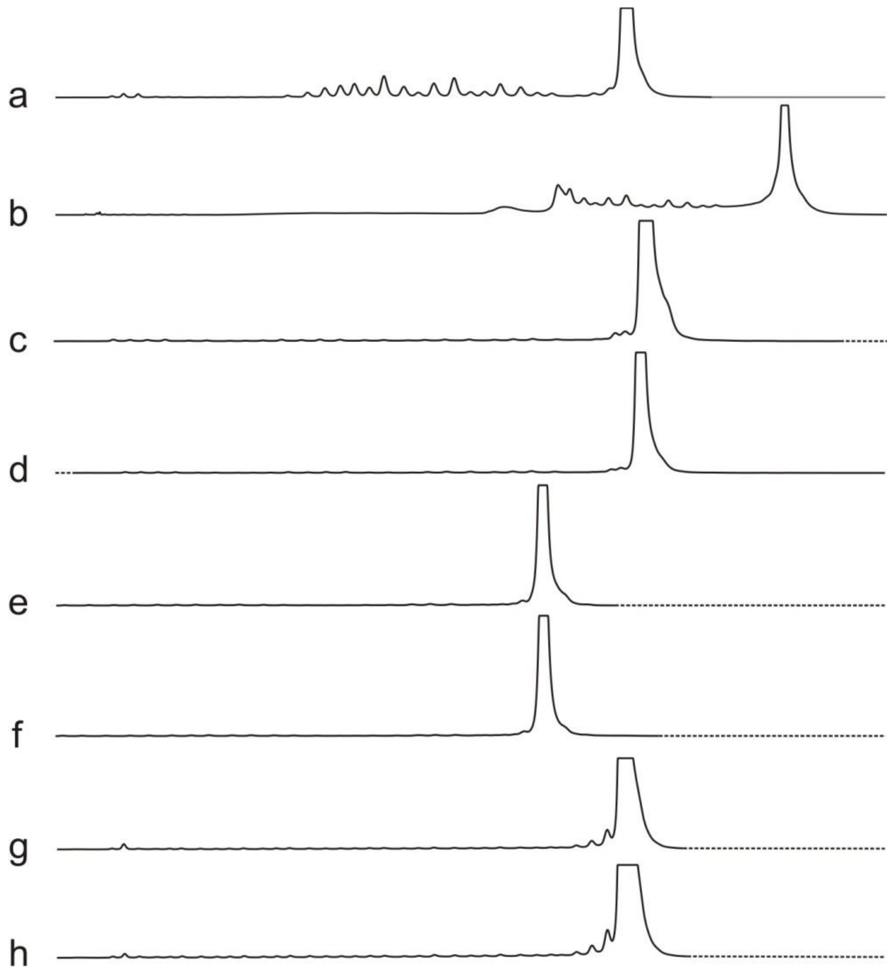

Figure S1: Additional tests for substrate specificities (150 nM substrate, 750 nM conjugate, 50 mM Tris-HCl, pH 8, 37 °C, 20 h).

Lane a: conjugate **11** and substrate **16** (complementary pair).

Lane b: conjugate **13** and substrate **16** (complementary pair).

Lane c: conjugate **11** and substrate **15** (not complementary).

Lane d: conjugate **13** and substrate **15** (not complementary).

Lane e: conjugate **11** and substrate **17** (not complementary).

Lane f: conjugate **13** and substrate **17** (not complementary).

Lane g: conjugate **12** and substrate **16** (not complementary).

Lane h: conjugate **14** and substrate **16** (not complementary).

This report was created by ACD/NMR Processor Academic Edition. For more information go to www.acdlabs.com/nmrproc/

Compound 7

19.11.2014 16:22:15

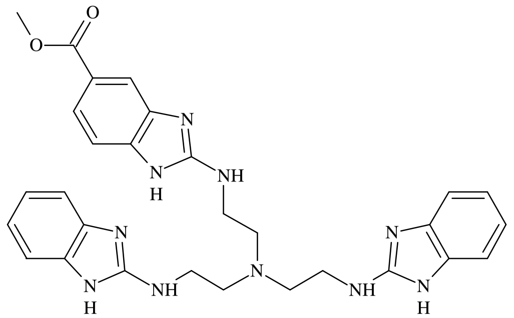

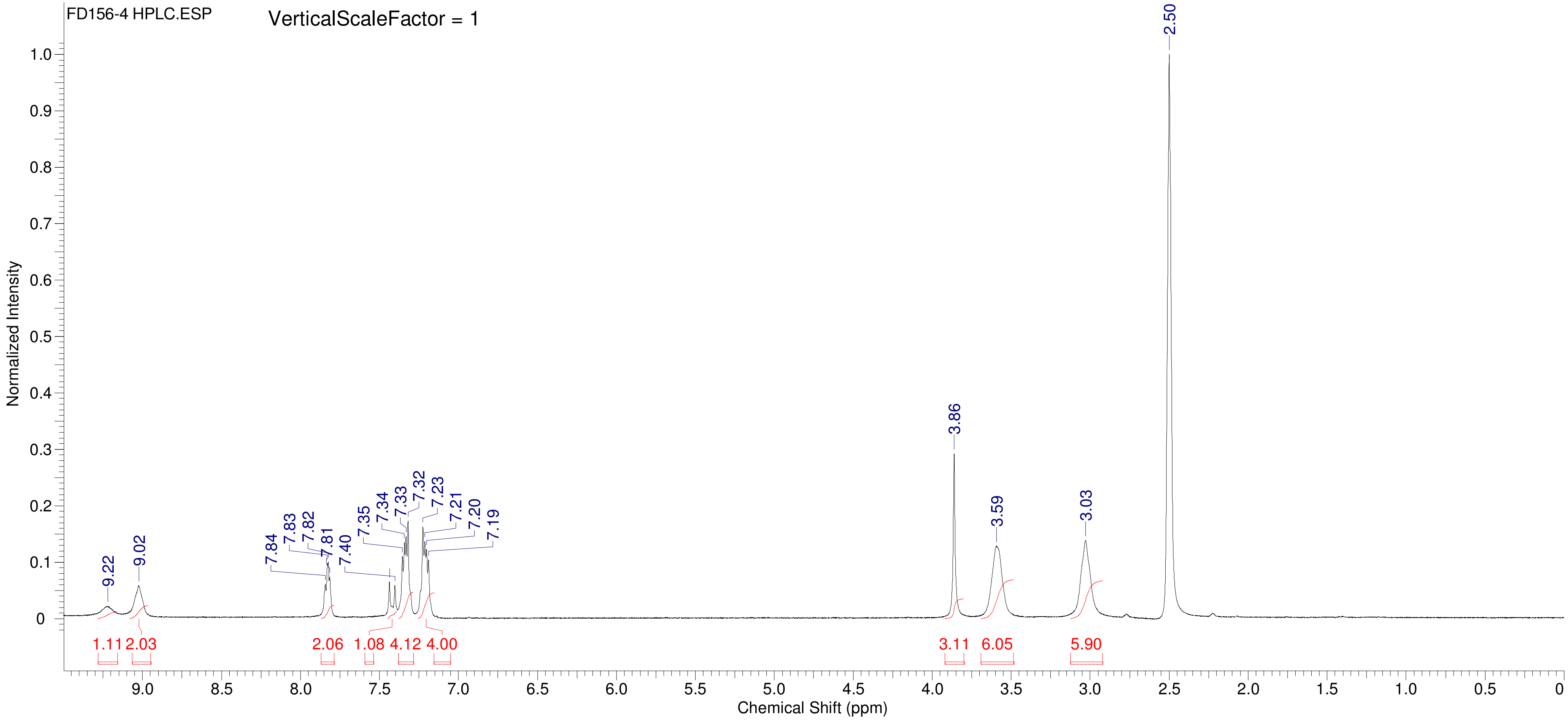

This report was created by ACD/NMR Processor Academic Edition. For more information go to www.acdlabs.com/nmrproc/

Compound 4

19.11.2014 16:36:40

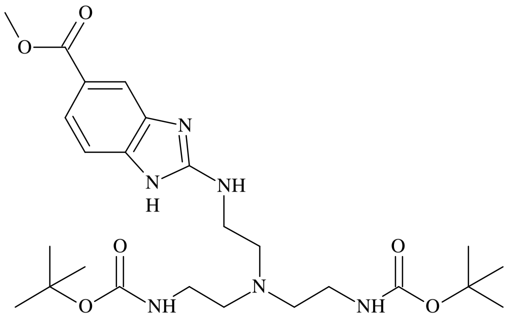

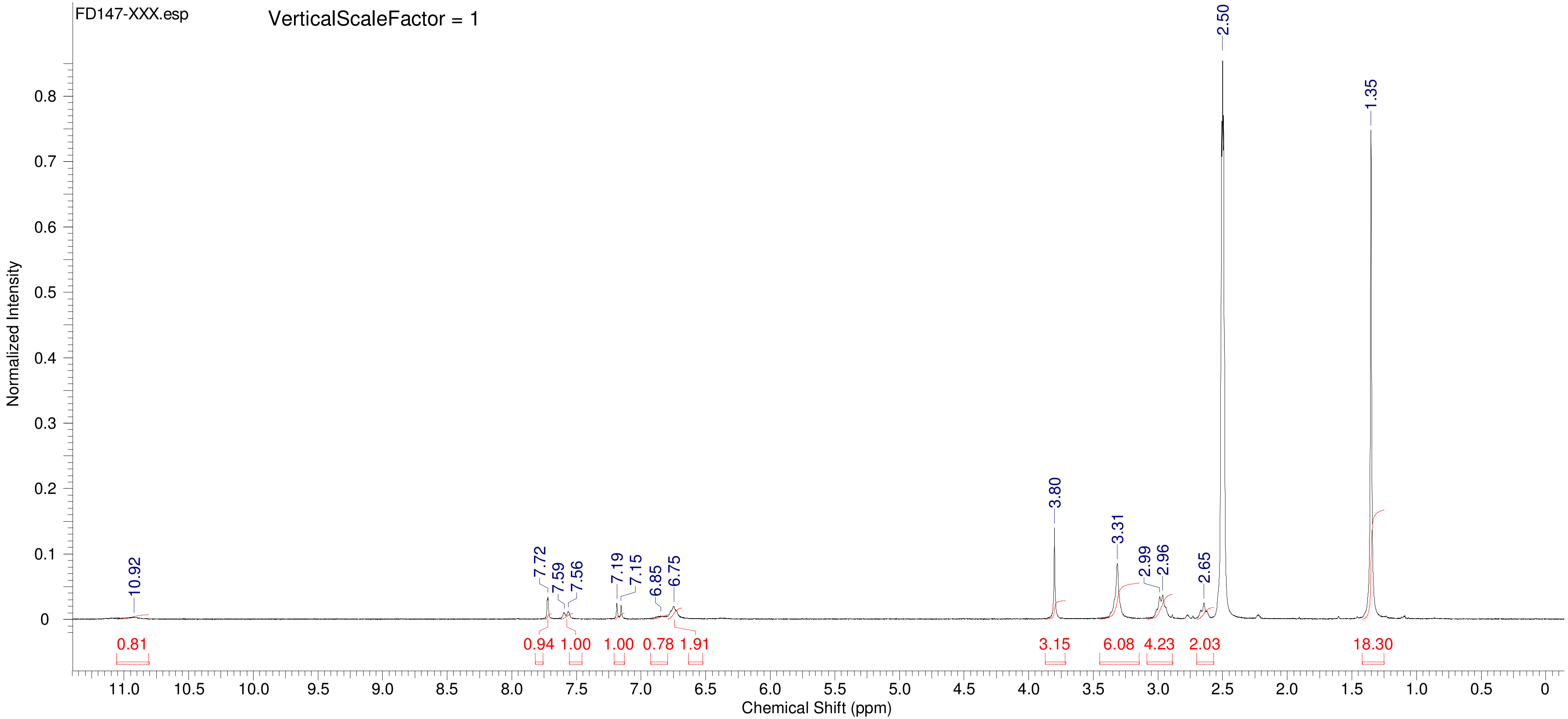

This report was created by ACD/NMR Processor Academic Edition. For more information go to www.acdlabs.com/nmrproc/

Compound 4

19.11.2014 16:38:33

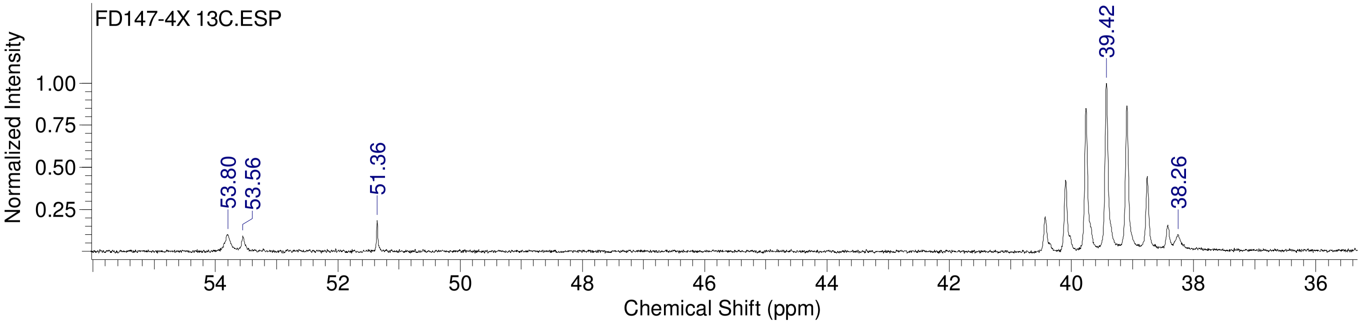

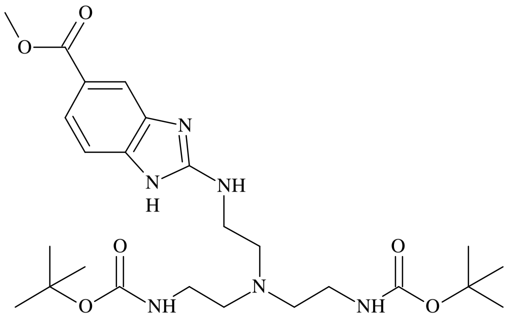

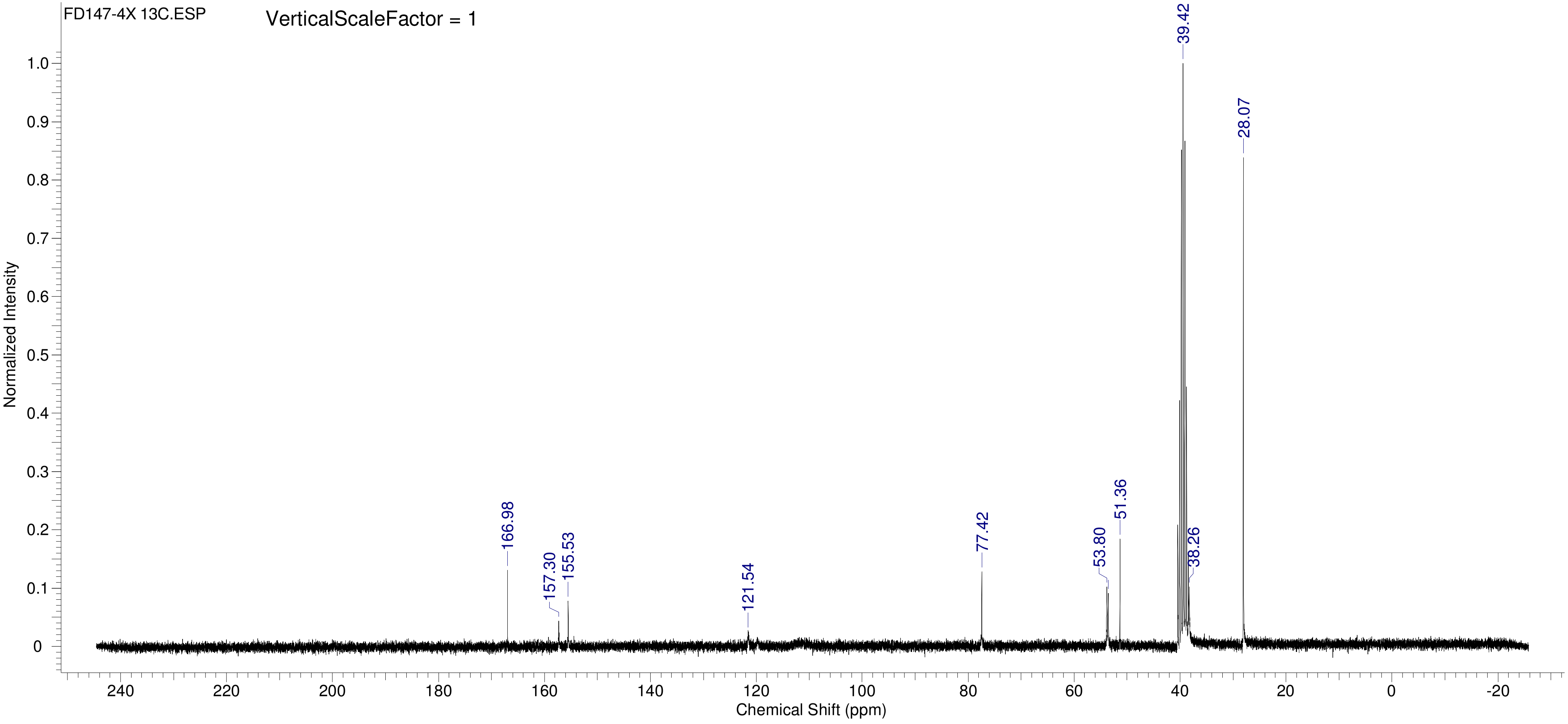

Supplement: File 1 — Experimental procedures and characterization data. [file Beilstein_J_Org_Chem-11-493-s001.pdf]
